# Poor Linkage to Care Despite Significant Improvement in Access to Early cART in Central Poland – Data from Test and Keep in Care (TAK) Project

**DOI:** 10.1371/journal.pone.0162739

**Published:** 2016-10-06

**Authors:** Justyna D. Kowalska, Leah Shepherd, Magdalena Ankiersztejn-Bartczak, Aneta Cybula, Hanna Czeszko-Paprocka, Ewa Firląg-Burkacka, Amanda Mocroft, Andrzej Horban

**Affiliations:** 1 Hospital for Infectious Diseases, HIV Out-Patient Clinic, Warsaw, Poland; 2 Medical University of Warsaw, Department for Adult's Infectious Diseases, Warsaw, Poland; 3 Department of Infection and Population Health, University College London, London, United Kingdom; 4 Foundation of Social Education (FES), Warsaw, Poland; 5 Medical University of Warsaw, Department of Infectious and Tropical Diseases and Hepatology, Warsaw, Poland; National and Kapodistrian University of Athens, GREECE

## Abstract

**Background:**

The main objective of the TAK project is investigating barriers in accessing HIV care after HIV-diagnosis at the CBVCTs of central Poland. Here we describe factors associated with and changes over time in linkage to care and access to cART.

**Method:**

Data collected in 2010–2013 in CBVCTs were linked with HIV clinics records using unique identifiers. Individuals were followed from the day of CBVCTs visit until first clinical visit or 4/06/2014. Cox-proportional hazard models were used to identify factors associated with being linked to care and starting cART.

**Results:**

In total 232 persons were diagnosed HIV-positive and 144 (62.1% 95%CI: 55.5–68.3) persons were linked to care. There was no change over time in linkage to care (p = 0.48), while time to starting cART decreased (p = 0.02). Multivariate factors associated with a lower rate of linkage to care were hetero/bisexual sexual orientation, lower education, not having an HIV-positive partner and not using condoms in a stable relationship. Multivariate factors associated with starting cART were lower education, recent year of linked to care, and first HIV RNA and CD4 cell count.

**Conclusions:**

Benefits of linkage to care, measured by access to early treatment, steadily improved in recent years. However at least 1 in 3 persons aware of their HIV status in central Poland remained outside professional healthcare. Persons at higher risk of remaining outside care, thus target population for future interventions, are bi/heterosexuals and those with lower levels of education.

## Introduction

It is estimated that up to 50% of HIV positive persons in Europe are unaware of their HIV infection and half of those newly diagnosed present late for care in Central and Eastern Europe [1–3]. Early diagnosis and initiation of combination antiretroviral therapy (cART) reduce morbidity and mortality to a significant degree, as well as reducing rates of onward HIV transmission and health care costs [4, 5].In order for this to occur, two crucial conditions must be met: firstly HIV should be diagnosed in a timely manner and secondly the patient should present to and be retained in medical care [6–8].

Increased HIV testing and strengthening of care pathways are encouraged in all settings. WHO and ECDC both recommend decentralization and diversification of HIV testing systems at both local and national levels [9]. This presents new challenges for linkage and engagement in HIV care, but there are few guidelines addressing this point [10]. Linkage to care remains an under investigated element in the continuum of care. Research into prompt linkage to and successful retention in HIV care in Central and Eastern Europe is limited, despite being highlighted as an important area for improving the quality of HIV care and containing the epidemic [11].

The Test and Keep in Care (TAK) project was initiated in response to the above, focusing on linkage to care of people who test HIV positive at three community-based voluntary counselling and testing facilities (CBVCTs) in central Poland. The main objective of the project is to estimate the proportion of HIV-positive persons linked and retained at each stage of HIV care and to investigate related factors in order to target effective interventions for linkage to care. The preliminary results indicated that significant proportion of patients were not linked to care [12]. Here we investigate factors associated with linkage to care and access to cART between 2010 and 2013 and describe changes in rates over time.

## Method

### Study methods

The detailed study methods were published elsewhere [12]. Briefly, data collected between 1/1/20110 and 31/12/2013 in three CBVCTs were linked with HIV clinics’ records using Western blot test number as the unique identifier. All persons tested HIV-positive receive detailed information about HIV specialist clinics where they can make an appointment over the phone or in person. Each person arriving at the clinic is seen by an HIV specialist immediately or at the time of his/hers choice and a personalized medical record is started. Persons registered in HIV clinics are followed routinely according to standards of medical care set by the European and Polish AIDS Societies [13].

Data collected in the study include pre-clinical data, which is self-reported by CBVCT clients on questionnaires standardised by National AIDS Centre. Information collected includes: nationality, level of education, sexual orientation, whether any HIV test had been performed previously and its location, number of tests within last year, year of test(s), details about sexual contacts, if ever tested for STI, hepatitis B and C, if partner was tested for HIV, if partner is HIV-positive, whether client was in a stable relationship within last year, condom use with stable partners and/or casual partners, number of stable and/or casual partners. This information is self-assessed by the CBVCT client, for example “stable relationship” reflects the client’s perspective and is not pre-defined. Clinical data included medical information collected by HIV clinics as part of routine healthcare.

### Patients and Inclusion criteria

People who were diagnosed HIV-positive in CBVCTs between 1/1/2010 and 31/12/2013 were included in the analysis. For the purpose of this study CBVCT clients who had a reactive HIV screening test (fourth generation test) were considered HIV-positive. Linkage to care was defined as date of first visit in the HIV clinic. Persons who tested HIV-positive, but failed to return to CBVCT for confirmatory Western-blot test, did not collected the confirmatory test or did not agree to de-coding of the confirmatory test were considered lost to care. Individuals were followed from the date of CBVCTs visit until earliest of first clinical visit or 4/06/2014.

Patients who were linked to care were further followed to determine whether they commenced cART (defined as receiving three or more antiretroviral drugs [ARVs]), from date of first clinical visit until date of first cART or 4/06/2014.

### Ethical approvals

The study has been approved by the Bioethical Committee of the Medical University of Warsaw (AKBE/99/16). Due to the anonymous character of CBVCT testing informed consent was not collected.

### Statistical analyses

Baseline characteristics of those who were linked to care and commenced cART were separately compared to those who were not using chi-squared test for categorical and Wilcoxon-Mann-Whitney for numerical variables. Kaplain-Meier estimates and log-rank tests were used to compare the rates of linkage to care and starting cART by year. Cox-proportional hazard models were used to identify factors associated with being linked to care (baseline date was HIV test date) and starting cART (baseline date was first visit in HIV clinic). Variables that were significant in the univariate analysis (defined as P-value <0.1) were adjusted for in the multivariate analysis. Variables considered for inclusion in both models were age at HIV test, gender, nationality, education, sexual orientation, test location, number of tests within last year, year of test, sexually transmitted infections, whether the partner was tested, whether partner was HIV-positive, if in a stable relationship within the last year, condom use with stable partners, condom use with casual partners, number of stable partners ever and in the last year, number of casual partners ever and in the last year. Additional variables considered for inclusion in the model for commencing cART; were measured at baseline and were Hepatitis B (HBV) and Hepatitis C (HCV) status, syphilis, HIV RNA level, lymphocyte CD4+ count, as well as CD3+, CD8+ and CD4+/CD8+ ratio. Correlation between clinical variables (HIV RNA level, CD4+ at first visit,CD3+, CD8+, CD4+/CD8+ ratio at first visit) were assessed using Spearman’s rho.

All statistical tests were two sided and a type I error rate of 5%. All statistical analyses were performed using SAS 9.3 (Statistical Analysis Software, Cary NC, USA).

## Results

In the study period, between 1/1/20110 and 31/12/2013, 232 persons were diagnosed HIV-positive in three CBVCTs. They were mostly Polish (96.1%), living in the central region of Poland (94.0%) and having higher education (77.6%). Among them 94.8% were male, 75.4% were men having sex with men (MSM), 22.8% had a HIV-positive partner, 11.6% were in a stable relationship and 36.2% had a partner who ever tested for HIV. Median age was 30.1 (IQR: 25.2–35.9) years.

### Linkage to care

**Fig 1:**
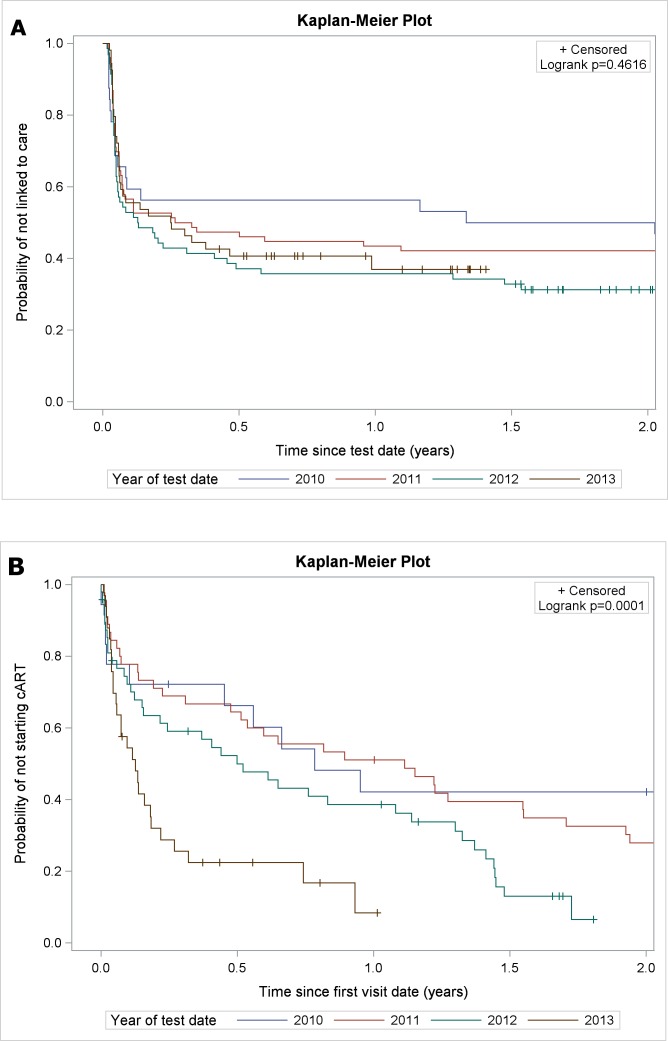
**a.** Kaplan-Meier plot for linked to care, stratified by year of HIV test **b.** Kaplan-Meier plot for starting cART by year of linked to care.

One hundred and forty four (62.1% with 95%CI: 55.5%–68.3%) persons were linked to care. Of those who were linked to care, 68.8% of linked patients registered within one month and 81.3% registered within three months from testing. There was no change over time in the proportion of persons linked to care (global p = 0.48) (Table 1, Fig 1A). The median time from the date of HIV test till first visit in HIV clinic was 0.6 (0.4–1.1) and till first lymphocyte CD4+ measurement was 0.6 (IQR: 0.4–1.0) months.

**Table 1:** Baseline pre-clinical characteristics for those who were linked to care and for those who started cART.

		*Linked to care*				*Started cART*		
*Factors*	*Total n (%)*	*no*	*yes*	*p-value*	*Total n (%)*	*no*	*yes*	*p-value*
***All***	232 (100)	88 (100)	144 (100)		144 (100)	27 (100)	117 (100)	
***Age at first visit***								
< = 30	116 (50.0)	50 (56.8)	66 (45.8)	0.10	63 (43.8)	12 (44.4)	51 (43.6)	0.94
>30	116 (50.0)	38 (43.2)	78 (54.2)		81 (56.3)	15 (55.6)	66 (56.4)	
***Sex***	*** ***	*** ***						
Male	220 (94.8)	83 (94.3)	137 (95.1)	0.78	137 (95.1)	25 (92.6)	112 (95.7)	0.50
Female	12 (5.2)	5 (5.7)	7 (4.9)		7 (4.9)	2 (7.4)	5 (4.3)	
***Nationality***								
Polish	223 (96.1)	83 (94.3)	140 (97.2)	0.28	140 (97.2)	27 (100)	113 (96.6)	0.98
Other	9 (3.9)	5 (5.7)	4 (2.8)		4 (2.8)	0 (0.0)	4 (3.4)	
***Education***								
high	180 (77.6)	63 (71.6)	117 (81.3)	0.09	117 (81.3)	22 (81.5)	95 (81.2)	0.97
lower than high/unknown	52 (22.4)	25 (28.4)	27 (18.8)		27 (18.8)	5 (18.5)	22 (18.8)	
***Sexual orientation***								
Homosexual	175 (75.4)	55 (62.5)	120 (83.3)	< .001	120 (83.3)	20 (74.1)	100 (85.5)	0.16
Bi/heterosexual	57 (24.6)	33 (37.5)	24 (16.7)		24 (16.7)	7 (25.9)	17 (14.5)	
***Test location***								
CBVCT	128 (55.2)	44 (50.0)	84 (58.3)	0.22	84 (58.3)	18 (66.7)	66 (56.4)	0.33
Other	104 (44.8)	44 (50.0)	60 (41.7)		60 (41.7)	9 (33.3)	51 (43.6)	
***Number of ests within last year***								
None	65 (28.0)	25 (28.4)	40 (27.8)	0.92	40 (27.8)	6 (22.2)	34 (29.1)	0.48
1+	167 (72.0)	63 (71.6)	104 (72.2)		104 (72.2)	21 (77.8)	83 (70.9)	
***Year of test***								
2010	32 (13.8)	14 (15.9)	18 (12.5)	0.57				
2011/10	76 (32.8)	31 (35.2)	45 (31.3)		60 (41.7)	11 (40.7)	49 (41.9)	0.94
2012	70 (30.2)	22 (25.0)	48 (33.3)		45 (31.3)	8 (29.6)	37 (31.6)	
2013	54 (23.3)	21 (23.9)	33 (22.9)		40 (27.1)	8 (29.6)	31 (26.5)	
***Sexually transmitted infection***								
No/unknown	197 (84.9)	77 (87.5)	120 (83.3)	0.39	120 (83.3)	20 (74.1)	100 (85.5)	0.16
Yes	35 (15.1)	11 (12.5)	24 (16.7)		24 (16.7)	7 (25.9)	17 (14.5)	
***Partner tested***								
No/unknown	148 (63.8)	56 (63.6)	92 (63.9)	0.97	92 (63.9)	20 (74.1)	72 (61.5)	0.23
Yes	84 (36.2)	32 (36.4)	52 (36.1)		52 (36.1)	7 (25.9)	45 (38.5)	
***Partner HIV+***								
No/unknown	179 (77.2)	75 (85.2)	104 (72.2)	0.02	104 (72.2)	18 (66.7)	86 (73.5)	0.48
Yes	53 (22.8)	13 (14.8)	40 (27.8)		40 (27.8)	9 (33.3)	31 (26.5)	
***In a stable relationship within last year***								
No/unknown	205 (88.4)	73 (83.0)	132 (91.7)	0.05	132 (91.7)	24 (88.9)	108 (92.3)	0.56
Yes	27 (11.6)	15 (17.0)	12 (8.3)		12 (8.3)	3 (11.1)	9 (7.7)	
***Condom use with stable partners***								
Yes	100 (43.1)	30 (34.1)	70 (48.6)	0.03	70 (48.6)	12 (44.4)	58 (49.6)	0.63
No/unknown	132 (56.9)	58 (65.9)	74 (51.4)		74 (51.4)	15 (55.6)	59 (50.4)	
***Condom use with casual partners***								
Yes	132 (56.9)	47 (53.4)	85 (59.0)	0.40	85 (59.0)	16 (59.3)	69 (59.0)	0.98
No/unknown	100 (43.1)	41 (46.6)	59 (41.0)		59 (41.0)	11 (40.7)	48 (41.0)	
***Number of stable artners***								
1–5,unknown	67 (28.9)	29 (33.0)	38 (26.4)	0.55	38 (26.4)	6 (22.2)	32 (27.4)	0.81
6–20	93 (40.1)	34 (38.6)	59 (41.0)		59 (41.0)	11 (40.7)	48 (41.0)	
>21	72 (31.0)	25 (28.4)	47 (32.6)		47 (32.6)	10 (37.0)	37 (31.6)	
***Number of casual partners***								
1–5,unknown	176 (75.9)	69 (78.4)	107 (74.3)	0.49	107 (74.3)	19 (70.4)	88 (75.2)	0.53
6–20	40 (17.2)	12 (13.6)	28 (19.4)		28 (19.4)	5 (18.5)	23 (19.7)	
>21	16 (6.9)	7 (8.0)	9 (6.3)		9 (6.3)	3 (11.1)	6 (5.1)	

Persons linked to care were o more likely to be MSM (83.3% vs. 62.5%; p<0.01), have an HIV-positive partner (27.8% vs. 14.8%;p = 0.02), to be using condom in a stable relationship (48.6% vs. 34.1%;p = 0.031) and less likely to remain in a stable relationship within last year (8.3% vs. 17.0%;p = 0.05) as compared to persons not linked to care. Other pre-clinical characteristics did not differ significantly (Table 1).

**Fig 2:**
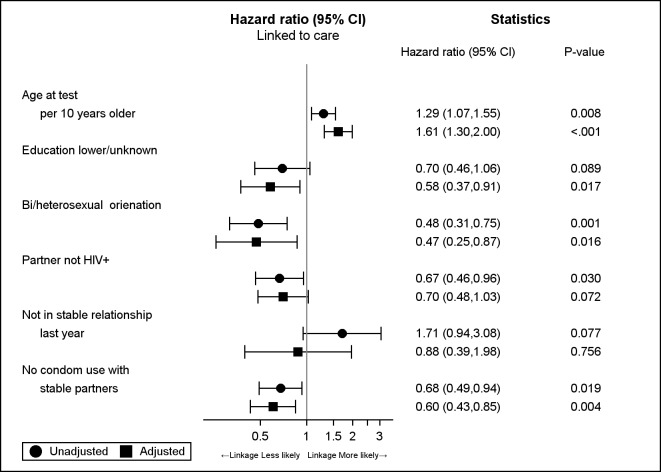
Adjusted and unadjusted hazard ratios for being linked to care in those tested for HIV at CBVTC.

In univariate models lower or unknown level of education (Hazard ratio [HR] = 0.70; 95% confidence interval [95%CI]: 0.46,1.06), bi- or heterosexual orientation (0.48; 0.31,0.75), having HIV-negative or HIV unknown status partner (0.67;0.46,0.96) and not using condoms with stable partner (0.68; 0.49,0.94) significantly decreased the chance of being linked to care. Older age was increasing the chance of being linked to care (HR per 10 years older: 1.29; 1.07,1.55) (Fig 2). After adjustment for age and factors with P<0.1, the chance of being linked to care was lower in those who were of bi- or heterosexual orientation (0.47; 0.25,0.87), lower or unknown level of education (0.58; 0.37,0.91) and not using condoms with stable partner (0.60; 0.43,0.85). Age remained the factor significant improving the likelihood of linkage by 61% per 10 years older (1.61; 1.30,2.00) (Fig 2). The median age at HIV test was 27.4 (IQR:24.2–34.1) years for those not linked and 30.4 (27.1–36.1) years for those linked to care. HIV status of partner, and being in a stable relationship were no longer significant after adjustment.

### Starting cART

Among the 144 people who were linked to care, 117 (81.3%) started cART during 118 person-years of follow up. The median follow-up per person was 5 (95% CI: 1–16) months.

The median age at first visit at the HIV clinic was 30.6 (IQR: 27.1,36.3) years. The median baseline lymphocyte CD3+ count was 1315 (1071, 1736) cells/μl, CD4+ count was 394 (292,507) cells/μl, CD8+ count was 850 (608,1182) cells/μl, median lymphocyte CD4+/CD8+ ratio was 0.4 (0.3,0.7) and HIV RNA 4.5 (3.9, 5.1) log10 copies/ml. In terms of co-infections 3 (2.1%) patients were anti-HCV positive, 16 (11.1%) of patients had HBV infection in the past but none had positive HBs antigen and 30 (20.8%) had positive Venereal Disease Research Laboratory test (VDRL) (Table 2).

**Table 2:** Additional baseline clinical characteristics for those who started cART.

	*Started cART*			
*Factors*	*Total n (%)*	*no*	*yes*	*p-value*
***HBV at first visit***				
Yes	16 (11.1)	5 (18.5)	11 (9.4)	0.40
No	103 (71.5)	18 (66.7)	85 (72.6)	
Unknown/missing	25 (17.4)	4 (14.8)	21 (17.9)	
***HCV at first visit***				
Yes	3 (2.1)	0 (0.0)	3 (2.6)	0.78
No	125 (86.8)	25 (92.6)	100 (85.5)	
Unknown/missing	16 (11.1)	2 (7.4)	14 (12.0)	
VDRL* ***test result at first visit***				
Yes	30 (20.8)	7 (25.9)	23 (19.7)	0.47
No/undetermined	100 (69.4)	19 (70.4)	81 (69.2)	
Unknown/missing	14 (9.7)	1 (3.7)	13 (11.1)	
***HIV VL at baseline***				
0–10,000	40 (27.8)	16 (59.3)	24 (20.5)	< .001
>10,000	104 (72.2)	11 (40.7)	93 (79.5)	
***Lymphocytes CD4+ at first visit (cells/μl)***				
1–350	49 (34.0)	2 (7.4)	47 (40.2)	0.005
351–500	58 (40.3)	12 (44.4)	46 (39.3)	
>500	37 (25.7)	13 (48.1)	24 (20.5)	
***Lymphocytes CD3+ at first visit (cells/μl)***				
Missing	1 (0.7)	0 (0.0)	1 (0.9)	0.30
1–1000	29 (20.1)	4 (14.8)	25 (21.4)	
1001–1500	63 (43.8)	9 (33.3)	54 (46.2)	
>1501	51 (35.4)	14 (51.9)	37 (31.6)	
***Lymphocytes CD8+ at first visit (cells/μl)***				
Missing	1 (0.7)	0 (0.0)	1 (0.9)	0.91
1–700	54 (37.5)	9 (33.3)	45 (38.5)	
701–1000	35 (24.3)	8 (29.6)	27 (23.1)	
>1000	54 (37.5)	10 (37.0)	44 (37.6)	
***Lymphocytes CD4+/CD8+ Ratio***				
Missing	1 (0.7)	0 (0.0)	1 (0.9)	0.013
0.01–0.3	47 (32.6)	2 (7.4)	45 (38.5)	
0.3–0.6	49 (34.0)	9 (33.3)	40 (34.2)	
>0.6	47 (32.6)	16 (59.3)	31 (26.5)	

* Venereal Disease Research Laboratory test

Pre-clinical characteristics were comparable between patients who started and not yet started cART. In terms of clinical characteristics, patients who started cART were more likely to have HIV RNA above 10 000 copies/ml (79.5% vs. 40.7%, p<0.01) and lymphocyte CD4+ count below 350 cells/μl (40.2% vs. 7.4%, p<0.01). They did not differ significantly in respect to VDRL, anti-HCV or anti-HBc total antibody statuses. There were 3 people who were HCV positive at baseline, all of whom started cART. Due to low numbers baseline HCV status was not considered for inclusion in cox models.

The time to starting cART decreased over calendar time and the percentage of patients who started cART within one year from their first visit in the clinic increased (Fig 1B).

In univariate models lower or unknown level of education (HR: 1.87; 95%CI: 1.16,3.01), more recent year of being linked to care (1.77; 1.12,2.80 for 2012, and 2.99; 1.79,4.99 for 2013/14), no condom use with casual partner (1.59; 1.08,2.33) and higher HIV RNA at baseline (HR per 10-fold higher: 1.79; 1.39,2.30) significantly increased the chance of starting cART. Chance of starting cART was lower in those with a higher number of casual partners (HR for >21 partners relative to 1–5 or unknown: 0.36; 0.15,0.87) and a higher CD4 count (0.69; 0.62,0.76). In univariate analysis, those with lower CD3+ lymphocyte count (HR per 2 fold higher: 0.61; 0.46,0.81) and CD4+/CD8+ (HR per 1 unit higher: 0.16; 0.07,0.36) ratio had lower chance of starting cART. However, these variables were not included in adjusted analyses due to high correlation with CD4+ lymphocyte count (Spearman’s rho: 0.54 and 0.66, respectively).

**Fig 3:**
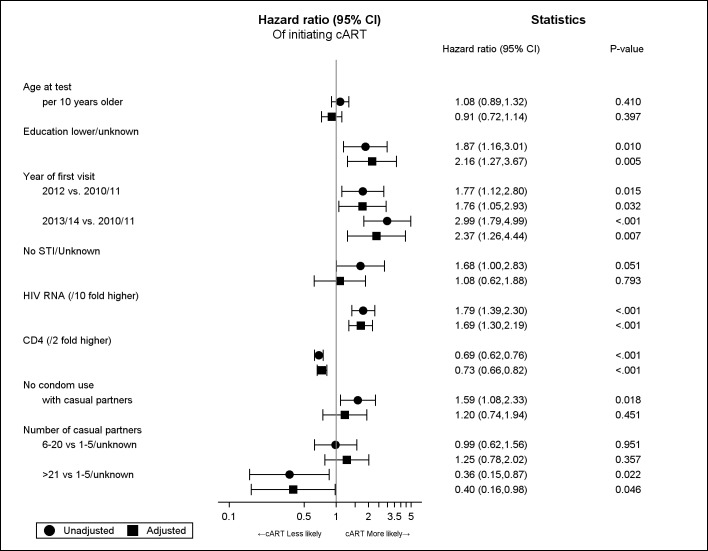
Adjusted and unadjusted hazard ratios for started cART in those who are tested.

After adjustment, only year of being linked to care, number of causal partners, baseline CD4+ count and baseline HIV RNA level remained significant. Persons linked to care in 2012 and in 2013/14 had respectively 76% and 137% higher chance of starting cART relative to those linked in 2010/11 (HR: 1.76; 1.05,2.93 and 2.37; 1.26,4.44—respectively). Persons with more than 20 casual partners had 60% lower chance of starting cART as compared to those having 5 or fewer lifetime partners (0.40; 0.16,0.98). The chance of starting cART was 69% higher for each 10 fold increase in HIV RNA (1.69; 1.30,2.19) and 27% lower for each doubling of CD4+ count (0.73; 0.66,0.82), Fig 3.

## Discussion

We have identified that 62.1% of people are linked to care after receiving a HIV diagnosis at CBVCTs in Central Poland and this has not improved over recent years. This proportion is lower than in Western European countries. For example in United Kingdom in 2011 the proportion of persons diagnosed and linked to care within one and three months were 88% and 97%, respectively [14]. Lower rates were reported in 2010 by seven autonomous regions of Spain where 75.7% of 1769 new HIV positive persons were linked to care within 3 months after diagnosis and 83.1% had evidence of linkage to care within a year [15]. High rates of linkage were reported among Portuguese MSM through an online survey with 97% of HIV positive respondents reporting visiting a health professional within 6 months from positive test result [16]. However in this survey most persons were tested in primary care settings, thus reflecting referral by a health professional to the HIV setting.

A comparison presented by Raymond et al. in 2014 confirmed large disparities in HIV treatment cascades across five European countries with the highest rate of linkage to care i.e. 81% in Denmark and the poorest i.e. 44% in Georgia [17–19].

However comparison of published data is difficult due to the lack of a standard definition of linkage to care. For example BHIVA guidelines recommend that newly diagnosed individuals should expect to have their HIV fully assessed by appropriately trained staff within 2 weeks of receiving an HIV-positive test result or to have CD4 result within one month [10]. Time till first CD4+ count test can be an accurate outcome measure for healthcare settings where such tests are immediately available. This may not be the case for low or middle income countries, therefore in 2011 WHO proposed a unified definition of linkage to care as duration of time from diagnosis to enrolment in HIV care or treatment [20]. However often countries use a combined definition e.g. timespan between documented clinical visit (CD4 cell count or HIV-1 viral load measurement) after HIV diagnosis, used by Chkhartishvili et al. [19].

Moreover the rates cannot be expected to be comparable for different settings. We found that 62.1% of persons tested anonymously at CBVCTs located outside medical facilities were linked to care, but this may not be representative of testing performed in medical settings. Moreover as observed by a pan-European project, namely the HIV-COBATEST, there is an important heterogeneity among CBVCTs [21]. It can be expected that rates of linkage to care after a positive HIV test at facilities which also test for other sexually transmitted diseases or are located at HIV clinics will be higher. However these facilities might be less frequented by people less open about their sexual orientation or behaviours.

In our study being bi- or heterosexual decreased the chance of being linked to care by approximately 50% and people with lower/unknown level of education by 40%, showing this is an focal group for future interventions, importantly as this group has been previously identified as more likely to be late presenters [2]. Factor increasing the chance of being linked to care was age, with 60% increase by each 10 years older. The median age at HIV test in our study was 30.1 years with 25% of clients being younger than 25.2 years. This implies younger clients should be consider at risk of not being linked.

In addition persons not using condoms with stable partner had a 40% lower chance of linkage, which is consistent with our previous findings, although the reasons are unclear [12].

In our study over 80% of persons linked to care subsequently started cART. Persons registered in the clinic in 2012 and 2013/4 had 76% and 137% respectively higher chances of starting cART relative to those registered in 2010/11. This suggests clinical decision making has been influenced by recent evidence from the HIV Prevention Trials Network (HPTN) Study 052 and START Study, supporting the benefit of cART initiation regardless of lymphocyte CD4+ count from both individual and population perspectives [4, 22]. It could also reflect a change in perception of the use of cART with easier to adhere and less toxic treatment alternatives [23, 24].

An interesting observation is the unexpected relationship between level of education and the chance of being linked to care and starting treatment. Persons with low/unknown level of education had a 42% lower chance of being linked to care, yet those who were linked to care had a 116% higher chance of starting cART. A large number of people who started cART had “unknown” education level, which prevented more detailed insight into this issue. However the observed discordance may reflect that persons from this group may struggle to be linked to care, but once being under physician care they follow advice more strictly. A similar pattern was previously described where persons with lower/middle level of education were less likely to have an HIV test [16].

Although only persons linked to care can fully benefit from being diagnosed, national surveillance systems rarely address this important issue in many countries. In response, in May 2015 WHO published the Strategic Information Guidelines for HIV in the healthcare system where “know your epidemic” is the most important first step into fighting the HIV epidemic. It is also highlighted that as much effort must be invested in linking/enrolling HIV-positive people into care as is put into scaling up HIV testing services [25]. This study is unable to investigate the impact of timely linkage to care and treatment initiation on outcomes. An important addition to current surveillance system in Poland would be the development of unique patient identifier/coding system which preserves anonymity while allowing for linkage of clinical data and outcome measures throughout the pathway of care.

There are some limitations which need to be mentioned. Firstly, we were not able to exclude the possibility of some patients linking to care in another city or region of Poland. As the central registration for new HIV infections in Poland is anonymous, we were not able to perform external auditing. The majority of patients in TAK study are from the central region of Poland and we rather see a trend towards centralization. Nevertheless we may have overestimated the number of persons not linked to care. Secondly, information on reasons for HIV testing was limited to sexual behaviours, which could have influence our understanding of possible barriers to linkage. Finally, the observed increase in cART uptake over time may be partially explained by the change in patients perception of treatment risks and benefits. We are not aware how many patients were offered cART and refused it and whether this has changed over time.

The majority of our study population were homosexual men, which may limit our ability to form conclusions on other risk groups due to small sample sizes. However our study population reflects the population of all HIV-positive persons in Poland and limited power due to low numbers of women or bi-sexual men will always be an issue [26].

Finally we based our study on routinely collected programmatic data, which has well recognised methodological limitations. On the other hand this data provide valuable insight into understanding the real-life scenario in public health, not only in Poland, but also in other Central European countries with comparable settings.

In response to earlier TAK results, all CBVTC in Poland started recording Western blot test numbers as unique identifier allowing for cross-checking with HIV clinics. It is very important to further integrate this pragmatic data into surveillance systems [27].

## Conclusions

There was no increase over time in linkage to care but the benefits of being linked to care, measured by access to early treatment, steadily improved in recent years. However, at least one in three persons aware of their HIV status in central Poland remained outside HIV care. Persons at higher risk of remaining outside care, thus target population for future interventions, are bi/heterosexuals with lower level of education.
